# Genome-wide characterization of the expansin gene family in *Sorghum bicolor* reveals their regulatory features and roles in drought response

**DOI:** 10.1186/s12870-026-09600-9

**Published:** 2026-07-30

**Authors:** Hameed Gul, Shareef Gul, Muhammad Usama, Sumit Singh, Muhammad Ibrahim, Ali Shahzad

**Affiliations:** 1 College of Agronomy and Biotechnology, Southwest University, Chongqing, 400715 China; 2 Institute of Horticultural Sciences, University of Agriculture Faisalabad, Faisalabad, 38040 Pakistan; 3 School of Breeding and Multiplication (Sanya Institute of Breeding and Multiplication), Hainan University, Sanya, Hainan 572025 China; 4 School of Tropical Agriculture and Forestry, Hainan University, Danzhou, 571737 China; 5 Department of Agriculture, University of Swabi, Swabi, KPK 23561 Pakistan

**Keywords:** Expansin gene family, *Sorghum bicolor*, Genome-wide analysis, Drought stress, Cell wall remodeling, qRT-PCR validation

## Abstract

**Background:**

Expansins (EXPs) are non-enzymatic proteins that mediate cell wall loosening and play essential roles in plant growth, development, and stress responses. However, the expansin gene family in *Sorghum bicolor* remains insufficiently characterized.

**Results:**

In this study, we identified 68 expansin genes in *S. bicolor* (*SbEXPs*), predominantly belonging to the EXPA and EXPB subfamilies. Structural and evolutionary characterization revealed conserved domain organization, with gene family expansion likely driven by tandem and segmental duplications. Regulatory analysis revealed diverse cis-regulatory elements and predicted interactions with transcription factors, miRNAs, and protein-protein interaction partners, indicating multi-layered regulatory control. Network analysis further identified key genes, including *SbEXPA17*,* SbEXPB1*, and *SbEXPB2*, as potential central nodes. Expression profiling demonstrated distinct tissue-specific patterns, with many *SbEXP* genes highly expressed in roots and other actively growing tissues. Under drought conditions, RNA-seq data revealed differential expression of several *SbEXP* genes, with a subset consistently upregulated. These patterns were validated by qRT-PCR, confirming strong induction of genes such as *SbEXPA17*,* SbEXPB6*, and *SbEXPB8* under PEG-simulated drought stress.

**Conclusion:**

This study provides a comprehensive characterization of the expansin gene family in *S. bicolor* and suggests their potential involvement in drought response through modulation of cell wall dynamics. These findings offer a foundation for future functional studies and the development of drought-resilient sorghum varieties.

**Supplementary Information:**

The online version contains supplementary material available at https://doi.org/10.1186/s12870-026-09600-9.

## Introduction

The growth and development of plants depend on the dynamic remodeling of the cell wall, a complex extracellular matrix composed predominantly of cellulose, hemicellulose, and pectin [1]. In addition to providing structural strength, the plant cell wall is a major interface that regulates cell expansion, intercellular communication, and responses to environmental stimuli [2]. This ability of plant cells to grow and adapt is thus strongly correlated with the regulated loosening and reorganization of this matrix [3]. Expansins (EXPs) are unique non-enzymatic proteins that facilitate cell wall loosening and extension, among the primary regulators of cell wall plasticity [4]. EXPs do not hydrolyze cell wall polysaccharides, unlike hydrolytic enzymes, but instead weaken the noncovalent binding between cellulose microfibrils and matrix polysaccharides, resulting in cell expansion under turgor [4, 5]. This unique mode of action underscores their central role in plant growth and morphogenesis.

The first proteins reported as EXPs were isolated from the hypocotyls of *Cucumis sativus*, and were capable of extending the cell wall in in vitro experiments without detectable enzymatic activity [6, 7]. Subsequent studies demonstrated that expansin activity is pH-dependent and closely associated with the acid growth theory, whereby the extrusion of protons lowers the apoplastic pH, enabling cells to loosen the cell wall [8]. Structurally, EXPs are generally composed of 250–275 amino acids and have two well-conserved domains: an N-terminal double-psi β-barrel (DPBB) domain and a C-terminal polysaccharide-binding domain [9]. The structural similarity between the DPBB domain and glycoside hydrolase family 45 proteins lies in their fold; however, the DPBB domain lacks catalytic activity, whereas the C-terminal domain facilitates binding to cell wall components [7, 9]. Based on sequence features and phylogenetic relationships, plant expansins are categorized into four subfamilies: α-expansin (EXPA), β-expansin (EXPB), expansin-like A (EXLA), and expansin-like B (EXLB). Among these, EXPA and EXPB are the most extensively studied and are primarily associated with cell elongation, organ development, and reproductive processes, whereas EXLA and EXLB members are more often linked to specialized functions and stress-related responses [10, 11].

Genome-wide analyses across many plant species have demonstrated that the EXP gene family is evolutionarily conserved but exhibits species-specific expansions, largely driven by tandem and segmental duplication events [11–13]. This expansion is often accompanied by functional diversification, enabling EXPs to participate in a variety of biological activities, including root and leaf growth, pollen tube growth, seed germination, fruit ripening, and fiber differentiation [14]. To illustrate, cell wall loosening regulated by EXPs is essential for root system architecture, enabling plants to effectively search the soil for water and nutrients, especially in stressful environments [8, 15]. In addition to their developmental roles, accumulating evidence indicates that *EXP* genes also contribute to plant responses to abiotic stresses, including drought, salinity, and extreme temperatures. These roles are often mediated through interactions with hormone signaling pathways, particularly abscisic acid (ABA), which coordinates growth and stress adaptation [16, 17]. Thus, EXPs serve as key components linking cell wall dynamics with environmental responsiveness.

In-depth analyses of the *EXP* gene family have revealed the diversification of *EXP* gene structure and function across multiple species. For instance, 46 *EXP* genes were identified in *Ginkgo biloba*, and most of them responded to hormonal and abiotic stress treatments [18]. *Salvia miltiorrhiza* has 29 *EXP* genes, and promoter analysis revealed a high abundance of stress- and hormone-responsive cis-regulatory elements [19]. Similarly, 30 *EXP* genes found in *yam (Dioscorea opposita)* showed evidence of segmental duplication-driven expansion and were associated with tuber development [20]. More than 59 *EXP* genes were identified as differentially expressed in *sweet potato* (*Ipomoea batatas*) in response to hormones and abiotic stresses, suggesting their involvement in both developmental and stress processes [21]. Moreover, recent genome- and transcriptome-scale analyses in Cucurbitaceae species have shown extensive expansion and functional diversification of *EXP* genes and their roles in plant adaptation and evolution [22].

Despite these advances, a comprehensive genome-wide characterization of the *EXP* gene family in *Sorghum bicolor* is still lacking. Sorghum is a major C4 cereal crop well known for its inherent tolerance to drought, salinity, and heat stress, making it an excellent model for studying stress adaptation mechanisms in crops [16, 23]. The ability to remain productive under water-limited conditions is associated with complex physiological and molecular mechanisms, including efficient water use, osmotic adjustment, and regulation of stress-responsive genes [24, 25]. Given the central role of EXPs in modulating cell wall plasticity, which is critical for both plant growth and stress adaptation, investigating the *EXP* gene family in sorghum is of particular importance.

Here, we carried out the genome-wide screening and integrated analysis of the *EXP* gene family in *S. bicolor*. To clarify their evolutionary and structural features, we have systematically examined their phylogenetic relationships, chromosome distributions, gene duplication patterns, conserved motifs, and cis-regulatory elements. In addition, transcriptomic profiling was combined with regulatory network analyses, including transcription factor and miRNA interactions, to uncover multilayered regulatory mechanisms controlling *SbEXP* genes. Lastly, publicly available RNA-seq data were analyzed to evaluate drought-responsive expression patterns, which were confirmed by qRT-PCR. This combined approach provides a novel understanding of the functional roles of *EXP* genes in sorghum and identifies highly valuable candidates for drought adaptation, providing important targets for enhancing crop performance in the future.

## Materials and methods

### Identification of *SbEXP* genes in *S. bicolor*

The genome datasets of *S. bicolor* (Sorghum_bicolor_NCBIv3) and *Zea mays* (Zm-B73-REFERENCE-NAM 5.0) were retrieved from the Ensembl Plants website (http://plants.ensembl.org/index.html), and for *Arabidopsis thaliana* (TAIR10), it was retrieved from TAIR website (http://www.arabidopsis.org). Members of the *SbEXP* gene family were identified using two complementary approaches. First, a BLASTP search was performed against the *S. bicolor* proteome using EXP protein sequences from *A. thaliana* as queries [26], with an E-value threshold of ≤ 1e⁻⁵ and a minimum alignment coverage of ≥ 50%. Second, HMMER (HMMsearch) was employed using Hidden Markov Models of the conserved expansin domains DPBB_1 (PF03330) and expansin_C (PF01357) obtained from the Pfam database (http://pfam.xfam.org), with the same E-value threshold (≤ 1e⁻⁵) [27, 28]. Non-redundant candidate sequences from both methods were merged and further validated for the presence of both conserved domains using PfamScan, NCBI Conserved Domain Database (CDD) (https://www.ncbi.nlm.nih.gov/Structure/cdd/wrpsb.cgi), and SMART (https://smart.embl.de/). Only proteins containing both DPBB_1 and expansin_C domains were retained for downstream analyses.

### Physicochemical properties and subcellular localization

Physicochemical properties of SbEXP proteins, including amino acid length, molecular weight (MW), isoelectric point (pI), and grand average of hydropathicity (GRAVY), were calculated using the ExPASy ProtParam tool (https://web.expasy.org/protparam/). Subcellular localization was predicted using CELLO (http://cello.life.nctu.edu.tw/).

### Phylogenetic analysis, gene structure, and conserved motifs

Multiple sequence alignment of SbEXP proteins along with *A. thaliana* EXP protein sequences was performed using ClustalW (https://www.genome.jp/tools-bin/clustalw). A phylogenetic tree was constructed using MEGA 11 based on the Neighbor-Joining (NJ) method with the JTT + I+G substitution model and 1,000 bootstrap replicates [29]. The resulting tree was visualized and annotated using iTOL v6.0 (https://itol.embl.de/) [30]. Both exon-intron patterns of the *SbEXP* genes were visualized using GSDS 2.0 (http://gsds.gao-lab.org) and TBtools [31]. Conserved motifs were identified using MEME Suite v5.5.3 (https://meme-suite.org/meme/), with motif width of 6-100 amino acids, a maximum of 15 motifs, and an E-value threshold of ≤ 1 × 10^− 10^ [32].

### Chromosomal distribution, gene duplication, and synteny analysis

Chromosomal locations of *SbEXP* genes were extracted from the sorghum genome annotation files and visualized using MapChart v2.32. Gene duplication events, including tandem and segmental duplications, were identified using MCScanX implemented in TBtools [33]. The synteny relationships between *S. bicolor* and *Z. mays* were analyzed and visualized using the Multiple Synteny Plotter and Advanced Circos modules in TBtools [34].

### Cis-acting regulatory element analysis

Promoter sequences (2,000 bp upstream of the transcription start site) of *SbEXP* genes were extracted using TBtools. Cis-regulatory elements were identified using the PlantCARE database (http://bioinformatics.psb.ugent.be/webtools/plantcare/html/) [35] and further validated using PLACE (https://www.dna.affrc.go.jp/PLACE/) and PlantPAN 3.0 (http://plantpan.itps.ncku.edu.tw/) [36]. Identified elements were categorized into functional groups, including light-responsive, hormone-responsive (e.g., ABRE, TGA-element, TCA-element), stress-responsive (e.g., MBS, LTR), and development-related elements. The distribution of these elements was visualized using TBtools.

### Protein–protein interaction (PPI) network and miRNA target prediction

To investigate the potential regulatory mechanisms of SbEXP proteins, we first predicted their protein–protein interactions (PPIs) using the STRING database (https://string-db.org/). Only interactions with a confidence score ≥ 0.4 (medium confidence), corresponding to the default STRING threshold, were retained to improve the reliability of the predicted interaction network. In addition, to explore the potential post-transcriptional regulation of *SbEXP* genes by miRNAs, we used the psRNATarget tool (https://www.zhaolab.org/psRNATarget/) to predict miRNA-target interactions [37]. The PPI network and miRNA interactions were visualized using Cytoscape v3.9.1 [38]. The interaction data were exported in TSV format and imported for further network analysis and visualization. This combined approach allowed us to explore potential regulatory relationships between SbEXP proteins and miRNAs, providing insights into their potential roles in stress-responsive gene regulation.

### Expression profiling of *SbEXP* genes

Expression profiles of *SbEXP* genes were analyzed using publicly available RNA-seq datasets from the NCBI Sequence Read Archive (SRA) (https://www.ncbi.nlm.nih.gov/sra) and the Phytozome Expression Atlas. Expression values (FPKM) were log₂-transformed as log2(FPKM + 1) to facilitate comparative analysis. Heatmaps representing gene expression across different tissues were generated using TBtools. For drought-specific analysis, RNA-seq data from the NCBI Gene Expression Omnibus (GEO) under accession number GSE128441 were utilized, as reported by Nazari and Zinati [38]. This dataset comprises transcriptomic profiles of *S. bicolor* under drought stress conditions, allowing assessment of differential gene expression in response to water deficit. Log₂ fold-change values as reported by Nazari and Zinati [38] were extracted from this dataset, and only *SbEXP* genes were retained for focused analysis, enabling assessment of their transcriptional responses to drought stress. We used log₂ fold-change values of this data set related to *SbEXP* genes.

### qRT-PCR validation and drought stress treatment

To study the expression of some *SbEXP* genes under drought, the seeds of *S. bicolor* (cv. BTx623) were used. Seeds were sterilized in 70% ethanol for 5 min, washed with sterile water, and placed on wet filter paper to germinate under controlled conditions (25 ± 2 °C, 16 h light/8 h dark cycle) [39]. Seedlings with three well-developed leaves were hydroponically grown and subjected to drought stress by treatment with 20% (w/v) polyethylene glycol (PEG 6000) in Hoagland’s nutrient solution. The control group (CK) was grown in a hydroponic solution without PEG [40]. Root tissues were harvested at 0 h (CK), 6 h, and 12 h after treatment, flash-frozen in liquid nitrogen, and stored at -80 °C for subsequent analysis.

Total RNA was isolated with TRIzol reagent (Invitrogen, Carlsbad, CA, USA) according to the manufacturer’s protocol. The concentration and purity of the RNA were assessed by a NanoDrop 2000 (Thermo Fisher Scientific, Wilmington, DE, USA). cDNA was synthesized with PrimeScript™ RT Reagent Kit (TaKaRa, Shiga, Japan). Quantitative real-time PCR (qRT-PCR) reactions were performed using SYBR Green Master Mix on an ABI7500 Real-Time PCR System (Applied Biosystems, Foster City, CA, USA). The housekeeping gene *SbACTIN* was used as an internal control. Three biological and three technical replicates were performed for each gene. Gene expression was quantified by the 2^⁻ΔΔCt^ method [41]. The sequences of primers used for qRT-PCR are provided in Supplementary Table S3.

### Data visualization and statistical analysis

All plots and analyses were generated in TBtools, GraphPad Prism 9.0, and Cytoscape 3.9.1, using default settings unless specified. For qRT-PCR, statistical significance was assessed by Student’s t-test with a threshold of *p* < 0.05.

## Results

### Genome-wide identification and characterization of *SbEXPs* in sorghum

To identify EXP proteins in *S. bicolor*, we employed both domain-based (HMM) and homology-based (BLASTP) approaches. The presence of the conserved DPBB_1 (PF03330) and expansin___C (PF01357) domains was verified using multiple domain prediction tools, and redundant sequences were removed. A total of 68 non-redundant EXP proteins were identified and designated as SbEXP1-SbEXP68 according to their chromosomal positions (Table 1; Supplementary Table S1). Phylogenetic classification grouped these proteins into four expansin subfamilies, including EXPA (37 members), EXPB (30 members), and a single EXLB member, while no EXLA proteins were detected. This distribution suggests that sorghum EXPs are predominantly represented by α- and β-expansin subfamilies. The identified SbEXP proteins exhibited considerable variation in their physicochemical properties. Protein lengths ranged from 196 to 325 amino acids, with an average of 269.9 aa. The predicted molecular weights ranged from 21.02 to 35.10 kDa, while isoelectric points ranged from 4.87 to 10.20, indicating diversity in protein charge. The GRAVY values ranged from − 0.476 to 0.147, suggesting that most SbEXP proteins are hydrophilic in nature. In addition, the instability index indicated that the majority of these proteins are stable or moderately stable in vitro. Subcellular localization prediction revealed that most SbEXP proteins (62 of 68) are localized to the extracellular region, which is consistent with their established roles in cell wall loosening and modification. A small number of proteins were predicted to localize to the chloroplast, mitochondrion, or cytoplasm, which may reflect functional diversification or limitations of prediction tools and warrants further experimental validation. Overall, the *SbEXP* gene family in sorghum appears to be moderately expanded and structurally conserved, with characteristics comparable to the EXP family reported in other plant species.

**Table 1:** A summary of detailed characteristics of the EXP gene family members in sorghum

Subfamily	Gene name	Gene ID	Transcript ID	Gene position	Length (aa)	MW (Da)	pI	Instability index	GRAVY	Subcellular localization
EXPA	*SbEXPA1*	*SORBI_3001G033300*	EER90621	1:2523358:2527858:1	262	28157.04	9.47	44.04	-0.053	Extracellular
	*SbEXPA2*	*SORBI_3001G237800*	EER91568	1:24405609:24407166:1	256	26947.38	8.41	37.29	0.066	Extracellular
	*SbEXPA3*	*SORBI_3001G237900*	EER91569	1:24426353:24428132:1	257	27445.27	9.04	27.9	0.083	Extracellular
	*SbEXPA4*	*SORBI_3001G238000*	EER94179	1:24430349:24431772:-1	253	26757.24	8.84	39.41	0.037	Extracellular
	*SbEXPA5*	*SORBI_3001G238100*	KXG38462	1:24465182:24466545:-1	253	26989.46	8.04	35.05	0.002	Extracellular
	*SbEXPA6*	*SORBI_3001G238200*	EER94181	1:24470313:24471749:-1	291	30917.84	7.96	37.7	0.016	Extracellular
	*SbEXPA7*	*SORBI_3001G238300*	EER91570	1:24476223:24477439:1	251	26,612	8.81	36.17	0.005	Extracellular
	*SbEXPA8*	*SORBI_3001G238400*	EER91571	1:24481079:24482375:1	253	26515.73	8.79	32.25	-0.035	Extracellular
	*SbEXPA9*	*SORBI_3001G314600*	EER94586	1:60271253:60272510:-1	264	28374.43	9.14	26.19	0.055	Extracellular
	*SbEXPA10*	*SORBI_3001G356400*	EER94786	1:64569251:64570347:-1	261	27445.09	10.2	52.28	0.026	Extracellular
	*SbEXPA11*	*SORBI_3001G499701*	OQU93207	1:76913420:76914768:-1	249	26647.03	9.03	31.3	-0.012	Extracellular
	*SbEXPA12*	*SORBI_3001G499800*	OQU93208	1:76917505:76925530:-1	261	27758.5	8.74	36.17	-0.009	Extracellular
	*SbEXPA13*	*SORBI_3001G499900*	EER95482	1:76927341:76928911:-1	254	27234.62	9.25	32.79	-0.139	Extracellular
	*SbEXPA14*	*SORBI_3002G305800*	KXG36242	2:68078457:68079892:1	272	29174.07	9.43	26.01	-0.446	Mitochondrial
	*SbEXPA15*	*SORBI_3003G059900*	EES02419	3:5295008:5296202:-1	253	26768.82	5.21	30.22	-0.078	Extracellular
	*SbEXPA16*	*SORBI_3003G112100*	EES02683	3:10081930:10084791:-1	254	26885.94	4.96	30.62	-0.069	Extracellular
	*SbEXPA17*	*SORBI_3003G128800*	EES00566	3:11875335:11883124:1	244	25194.12	8.08	31.75	-0.002	Extracellular
	*SbEXPA18*	*SORBI_3003G338801*	OQU87763	3:66191754:66194027:1	252	26440.47	6.38	36.31	-0.081	Extracellular
	*SbEXPA19*	*SORBI_3003G444400*	EES02147	3:74266256:74267531:1	282	30199.46	9.67	38.3	-0.039	Extracellular
	*SbEXPA20*	*SORBI_3004G119600*	EES04897	4:12817997:12819125:1	257	27516.49	9.46	27.45	0.027	Extracellular
	*SbEXPA21*	*SORBI_3004G119800*	EES04898	4:12833400:12834729:1	270	28760.83	9.16	31.17	-0.05	Extracellular
	*SbEXPA22*	*SORBI_3004G121600*	EES04906	4:13330906:13332319:1	262	28287.34	9.22	32.53	-0.09	Extracellular
	*SbEXPA23*	*SORBI_3004G121700*	EES04907	4:13374207:13375247:1	255	27558.53	9.39	30.09	-0.103	Extracellular
	*SbEXPA24*	*SORBI_3004G121800*	EES04908	4:13392150:13393497:1	259	27870.91	9.02	31.33	-0.014	Extracellular
	*SbEXPA25*	*SORBI_3004G121900*	EES04909	4:13420736:13422191:1	258	27784.79	9.13	27.78	-0.032	Extracellular
	*SbEXPA26*	*SORBI_3004G238801*	OQU85427	4:58664711:58666648:-1	292	30350.99	9.43	35.59	-0.114	Extracellular
	*SbEXPA27*	*SORBI_3006G031900*	EES10521	6:6888717:6890214:1	263	27951.42	8.63	37	-0.081	Extracellular
	*SbEXPA28*	*SORBI_3006G191700*	EES11318	6:54588520:54591502:1	257	27832.95	8.53	35.72	-0.018	Extracellular
	*SbEXPA29*	*SORBI_3007G018000*	EES14420	7:1554396:1555756:-1	266	28408.89	5.63	36.31	-0.11	Extracellular
	*SbEXPA30*	*SORBI_3007G019900*	EES14433	7:1855603:1856772:-1	278	30053.05	7.47	45.58	0.022	Extracellular
	*SbEXPA31*	*SORBI_3007G020100*	EES13270	7:1873666:1875062:1	260	28103.82	6.24	35.84	0.048	Extracellular
	*SbEXPA32*	*SORBI_3007G020200*	EES13271	7:1885830:1887792:1	259	27987.69	6.24	36.12	0.052	Extracellular, Lysosomal
	*SbEXPA33*	*SORBI_3007G166500*	KXG25373	7:60163042:60163905:1	274	28829.68	9.11	36.1	-0.106	Extracellular
	*SbEXPA34*	*SORBI_3009G173700*	EES19693	9:52914266:52924065:-1	248	25831.78	6.87	35.11	-0.077	Extracellular
	*SbEXPA35*	*SORBI_3010G006200*	EER89054	10:503695:504961:-1	276	29254.15	8.51	42.92	0.054	Extracellular
	*SbEXPA36*	*SORBI_3010G194500*	EER88662	10:53707267:53710736:1	261	28096.86	6.88	28.4	-0.024	Extracellular
	*SbEXPA37*	*SORBI_3010G268700*	KXG20914	10:60323587:60324852:1	270	28629.26	9.53	39.24	-0.06	Extracellular
EXPB	*SbEXPB1*	*SORBI_3001G155600*	OQU91274	1:12596116:12599103:-1	325	34230.69	4.96	37.35	0.095	Extracellular
	*SbEXPB2*	*SORBI_3001G155700*	EER93754	1:12601267:12605527:-1	306	32126.19	5.34	33.15	0.147	Extracellular
	*SbEXPB3*	*SORBI_3001G300400*	EER94500	1:58170939:58174678:-1	290	31852.82	6.99	30.22	-0.406	Extracellular
	*SbEXPB4*	*SORBI_3001G300500*	KXG38933	1:58200893:58202638:1	319	35099.67	8.68	30.73	-0.255	Extracellular
	*SbEXPB5*	*SORBI_3001G300700*	EER91942	1:58217949:58219755:1	266	29126.6	5.35	27.95	-0.233	Extracellular
	*SbEXPB6*	*SORBI_3001G300800*	EER91943	1:58236575:58238354:1	266	28805.24	4.87	27.37	-0.207	Extracellular
	*SbEXPB7*	*SORBI_3001G300900*	OQU92157	1:58244005:58250989:1	308	33463.73	5.75	27.15	-0.086	Extracellular
	*SbEXPB8*	*SORBI_3001G301000*	EER91945	1:58262947:58264636:1	276	29946.98	9.62	36.77	-0.215	Extracellular
	*SbEXPB9*	*SORBI_3001G301300*	EER91947	1:58289455:58290954:1	269	28920.15	9.51	27.41	-0.149	Extracellular
	*SbEXPB10*	*SORBI_3001G301400*	EER91948	1:58302321:58303918:1	278	29996.87	9.56	28.48	-0.242	Extracellular
	*SbEXPB11*	*SORBI_3001G301500*	EER91949	1:58340492:58342011:1	282	30885.74	7.02	36.84	-0.295	Extracellular
	*SbEXPB12*	*SORBI_3001G301600*	EER94506	1:58350449:58351958:-1	282	30853.7	6.64	37.67	-0.294	Extracellular
	*SbEXPB13*	*SORBI_3001G306200*	KXG38969	1:59017976:59019119:-1	266	28693.17	9.15	25.79	-0.175	Extracellular
	*SbEXPB14*	*SORBI_3001G306400*	EER94537	1:59040123:59041559:-1	266	28753.2	9.15	28.33	-0.222	Extracellular
	*SbEXPB15*	*SORBI_3001G306500*	KXG38972	1:59118001:59119187:-1	266	28741.15	9.15	28.7	-0.242	Extracellular
	*SbEXPB16*	*SORBI_3001G539760*	OQU93456	1:80254494:80255132:1	212	23088.26	7.69	25.8	-0.476	Cytoplasmic
	*SbEXPB17*	*SORBI_3001G542100*	KXG40416	1:80531707:80533460:-1	320	33849.92	5.97	43.27	-0.34	Extracellular
	*SbEXPB18*	*SORBI_3001G542200*	OQU93475	1:80544367:80545961:1	273	29865.78	9.04	34.76	-0.274	Extracellular
	*SbEXPB19*	*SORBI_3002G124400*	EER98447	2:16611974:16613474:-1	307	31962.64	9.44	37.42	-0.066	Chloroplast
	*SbEXPB20*	*SORBI_3002G124500*	EER98449	2:16648054:16649441:-1	312	33031.05	9.55	40.49	-0.19	Chloroplast
	*SbEXPB21*	*SORBI_3004G191600*	OQU85199	4:54338688:54341686:1	296	32262.41	6.15	39.31	-0.163	Extracellular
	*SbEXPB22*	*SORBI_3004G227900*	OQU85377	4:57749440:57753429:1	196	21017.96	9.34	31.77	-0.099	Extracellular
	*SbEXPB23*	*SORBI_3004G294300*	EES05780	4:63429826:63431751:1	295	30770.99	8.84	37.14	-0.069	Extracellular
	*SbEXPB24*	*SORBI_3004G294400*	EES07508	4:63449764:63451513:-1	294	30766.06	8.82	41.96	-0.067	Extracellular
	*SbEXPB25*	*SORBI_3004G294500*	EES07509	4:63460123:63462394:-1	264	27406.98	7.49	41.83	0.034	Extracellular
	*SbEXPB26*	*SORBI_3006G171100*	EES11233	6:52761527:52763240:1	288	30560.9	9.87	33.88	-0.065	Extracellular
	*SbEXPB27*	*SORBI_3006G171200*	EES11234	6:52765817:52767437:1	263	26909.39	7.5	29.2	0.13	Extracellular
	*SbEXPB28*	*SORBI_3006G171300*	EES11235	6:52770941:52773652:1	291	30693.6	9.6	37.6	-0.173	Extracellular
	*SbEXPB29*	*SORBI_3010G120100*	EER88254	10:13680107:13681549:1	259	26903.23	7.45	37.44	-0.029	Extracellular
	*SbEXPB30*	*SORBI_3010G121800*	KXG19818	10:14150586:14152163:-1	259	26757.94	6.79	36.93	-0.047	Extracellular
EXLB	*SbEXLB1*	*SORBI_3001G516500*	EER95568	1:78317740:78320162:-1	270	29211.45	9.03	32.85	-0.021	Extracellular, Plasma Membrane

### Phylogenetic analysis of *SbEXP* genes

**Fig. 1:**
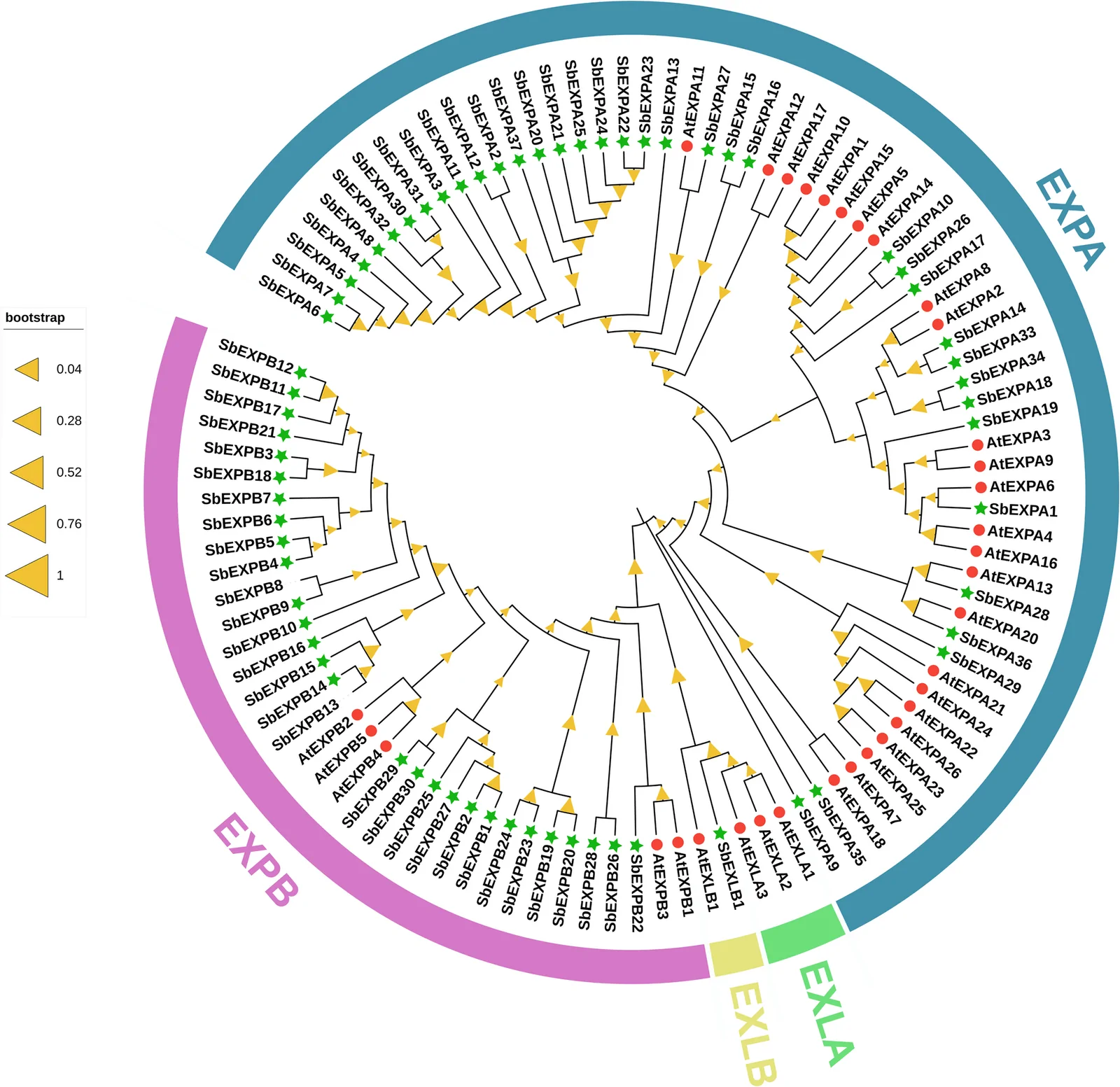
Phylogenetic tree of expansins from *Sorghum bicolor* and *Arabidopsis thaliana* constructed using SbEXP and AtEXP proteins and visualized in iTOL

To investigate the evolutionary relationships among the EXP proteins in *S. bicolor*, a Neighbor-Joining tree was constructed using all identified SbEXP sequences, together with EXPs from *A. thaliana*, with 1,000 bootstrap replicates (Fig. 1). The tree classified the proteins into the four established expansin subfamilies: EXPA, EXPB, EXLA, and EXLB. As identified (Section "Genome-wide identification and characterization of *SbEXPs* in sorghum"), the majority of SbEXPs were grouped within the EXPA and EXPB subfamilies, while only one SbEXLB member was identified, and no SbEXLA proteins were detected. Several Sorghum-specific clusters were observed, where SbEXP proteins grouped together with short branch lengths. Such clustering most likely reflect lineage-specific diversification, possibly driven by recent gene duplications. In addition, some SbEXP proteins showed close relationships to Arabidopsis EXPs, suggesting possible functional conservation. The uneven distribution of *SbEXP* genes across subfamilies, characterized by the expansion of EXPA and EXPB and the absence of EXLA members, further supports a monocot-specific pattern of EXP family evolution. In summary, the phylogenetic analysis shows that *SbEXP* genes are conserved and have also undergone lineage-specific expansion, suggesting possible functional diversification in sorghum.

### Gene structure and conserved motif analysis of *SbEXP* genes

**Fig. 2:**
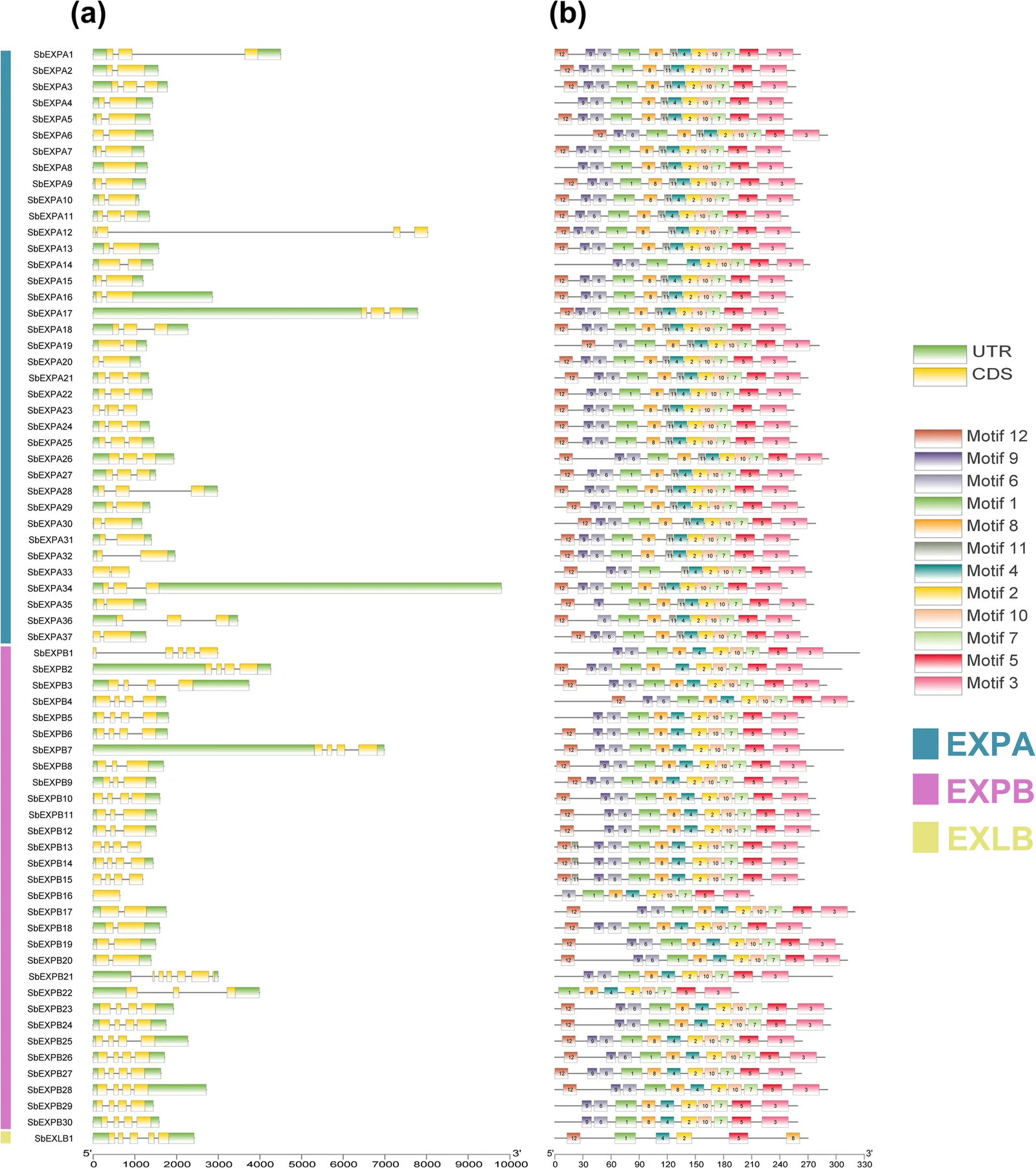
Motif and gene structures of expansin genes in *Sorghum bicolor*. **a** Gene structure of *SbEXP* genes, where yellow boxes represent coding sequences (CDS), green boxes are untranslated regions (UTRs), and lines are introns. **b** Colored boxes represent conserved motifs predicted by MEME in protein sequences. Subfamily grouping (EXPA, EXPB, EXLB) is shown

To gain insight into the structural properties of the *SbEXP* gene family members, exon–intron organization and conserved motifs were analyzed (Fig. 2). Structural analysis revealed clear subfamily-specific patterns. *EXPA* genes exhibited a relatively conserved exon–intron structure, containing two to five exons, whereas *EXPB* genes showed greater variability, with exon numbers ranging from one to eight. The single *EXLB* member displayed a seven-exon structure, consistent with previous reports in other plant species. While untranslated regions (UTRs) showed considerable variation in length among genes, the predicted coding sequences within each subfamily were relatively conserved, indicating structural constraints on functional regions. In some cases, longer intronic or UTR regions were observed, which may reflect regulatory complexity rather than alterations in protein structure. A total of twelve conserved motifs were identified across SbEXP proteins (Fig. 2). Motifs corresponding to the N-terminal DPBB_1 domain and the C-terminal expansin_C domain were present in nearly all members, highlighting their essential roles in expansin function. However, other motifs showed subfamily-specific conservation, especially among EXPB members, in which additional motifs were frequently located in the C-terminal region of the protein. The combination of conserved core motifs and subfamily-specific features suggests that SbEXP proteins maintain fundamental structural functions while allowing diversification in regulatory or interaction potential. In addition, the consistency between phylogenetic relationships (Section "Phylogenetic analysis of *SbEXP* genes") and gene structure and motif organization supports both the evolutionary conservation and functional differentiation of *SbEXP* genes.

### Chromosomal distribution and duplication dynamics of *SbEXPs*

**Fig. 3:**
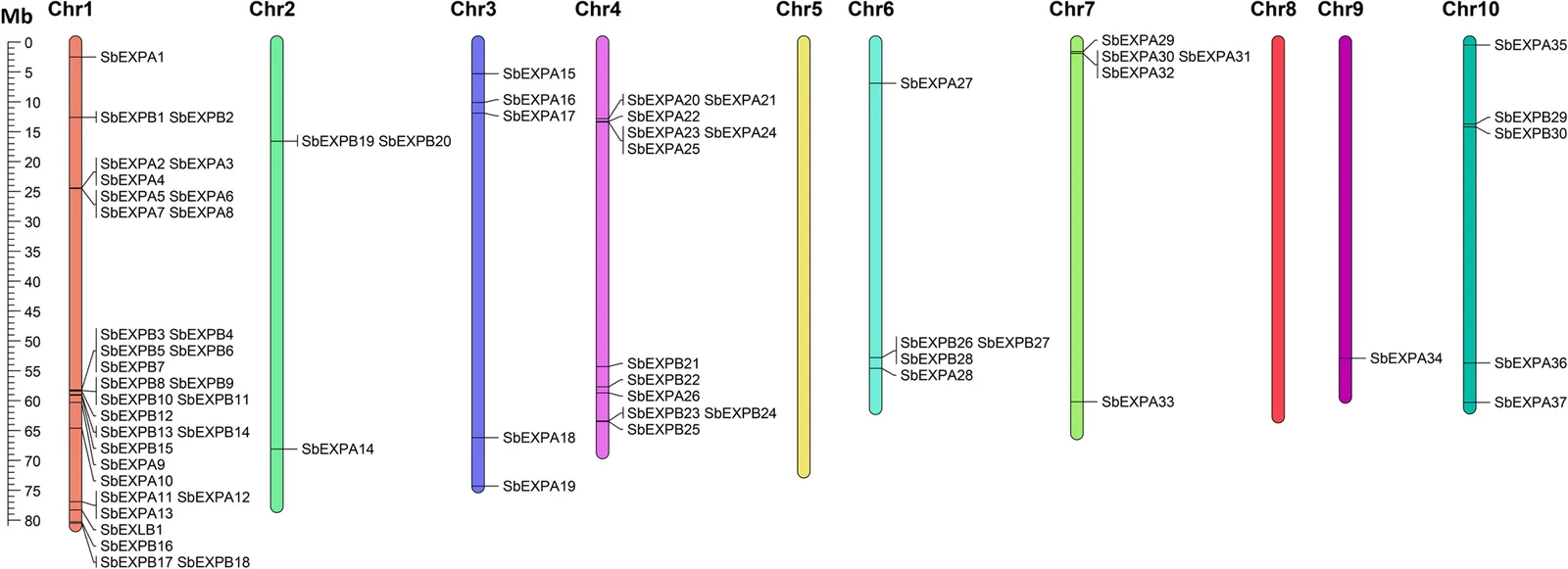
Chromosomal distribution of *SbEXP* genes on the ten *Sorghum bicolor* chromosomes

**Fig. 4:**
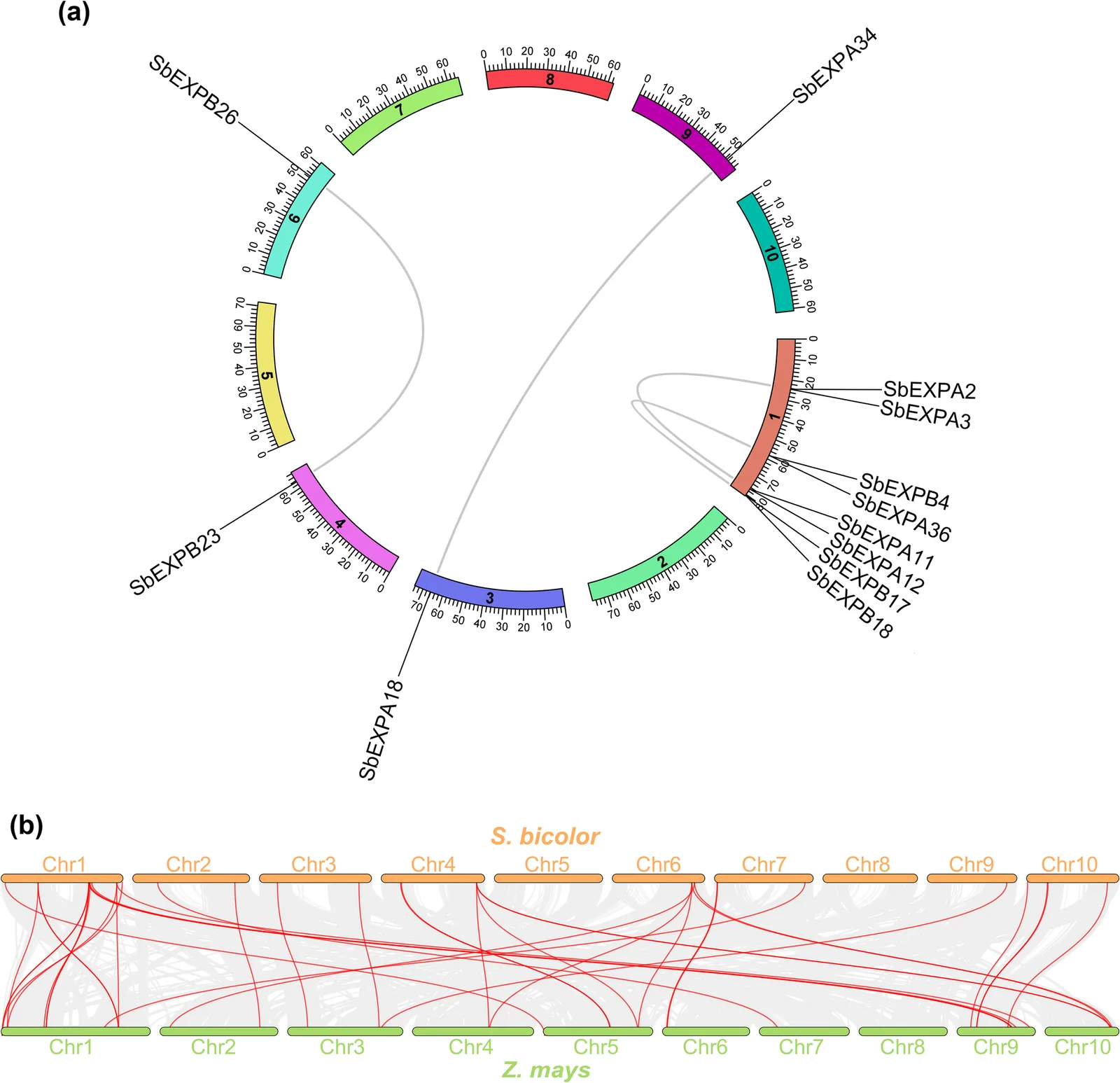
**a** Intra-chromosomal collinearity of segmentally duplicated *SbEXP* genes. **b** Synteny between *Sorghum bicolor* and *Zea mays* showing conserved expansin gene blocks. Duplicated or collinear gene pairs are joined by lines

Mapping of the 68 *SbEXP* genes onto the *S. bicolor* chromosomes revealed an uneven distribution (Fig. 3). The majority of genes were located on chromosomes 1 (32 genes) and 4 (12 genes), while fewer were distributed across chromosomes 2, 3, 6, 7, 9, and 10. No *SbEXP* genes were detected on chromosomes 5 and 8. This distribution pattern suggests region-specific expansion of the *SbEXP* gene family within the sorghum genome. To further explore the evolutionary dynamics of this gene family, duplication analysis was performed. Whole genome duplication (WGD) or segmental duplication events were identified, indicating their contribution to the expansion of the *SbEXP* gene family (Fig. 4a). Synteny analysis between *S. bicolor* and *Z. mays* revealed numerous conserved orthologous gene pairs, particularly within the EXPA and EXPB subfamilies (Fig. 4b). The conservation of these collinear gene pairs suggests that expansin genes have been preserved across grass genomes. Overall, these results indicate that the *SbEXP* gene family has expanded through both tandem and segmental duplication events and retains conserved features across related species.

### Cis-regulatory element composition of *SbEXP* promoters

**Fig. 5:**
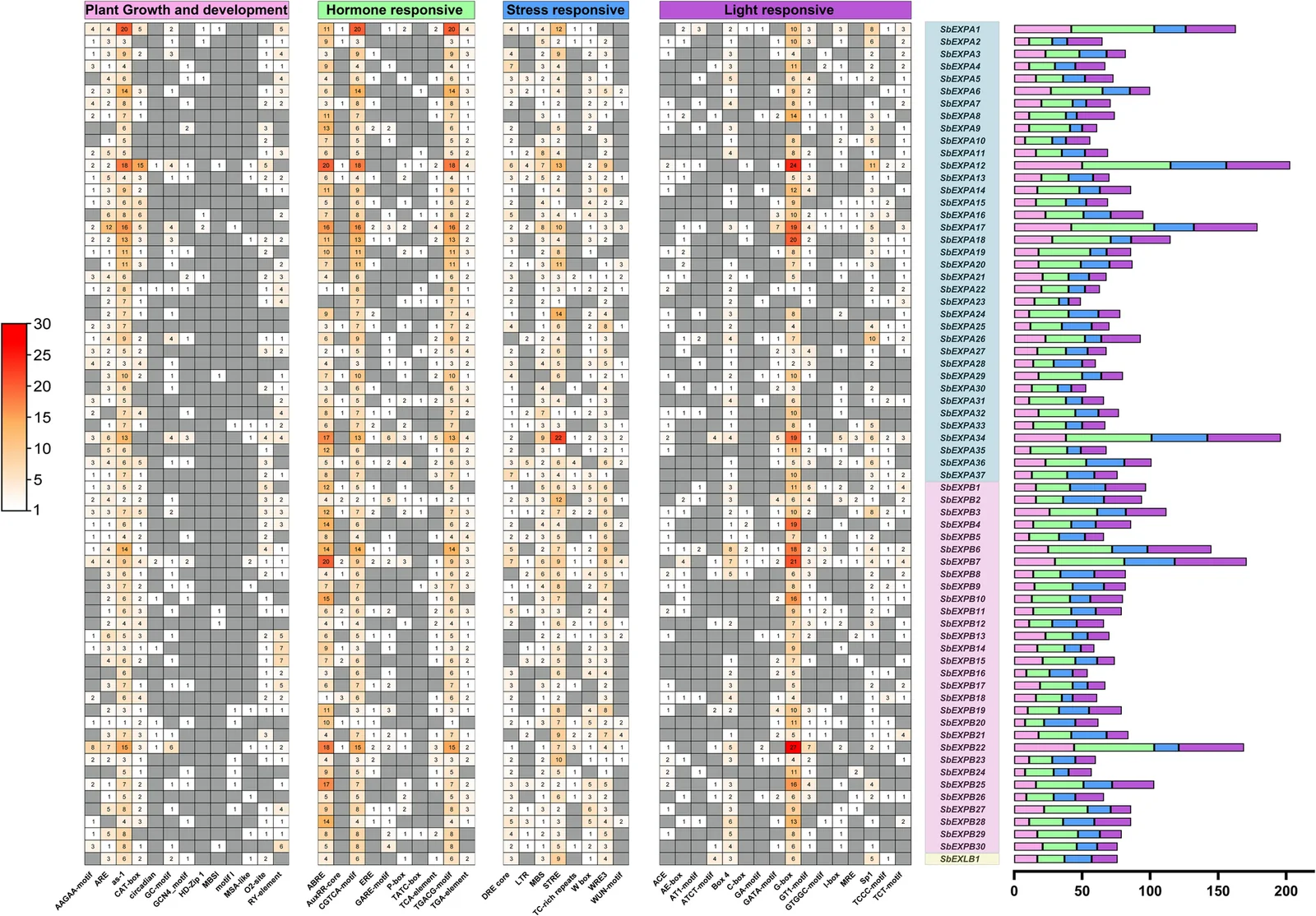
Cis-elements in *SbEXP* promoters. Heat map of cis-elements in 2 kb upstream promoter regions of *SbEXP* genes presented as four categories: plant growth and development, hormone responsiveness, stress responsiveness, and light responsiveness. The stacked bar represents the total number of cis-elements. Shades represent the abundance of the motifs

A wide range of cis-regulatory elements associated with plant growth and development were identified in the 2-kb upstream promoter regions of *SbEXP* genes, including hormone-, stress-, light-, and development-related elements, which were unevenly distributed (Fig. 5; Supplementary Table S2). Core promoter elements such as TATA-box and CAAT-box were present in almost all genes, indicating the integrity of the analyzed promoter regions. Hormone-responsive elements were abundantly represented in *SbEXP* promoters. In particular, abscisic acid (ABA)-responsive elements (ABREs) were highly enriched, indicating potential regulation through ABA signaling pathways. Cis-elements related to auxin (TGA-element, AuxRR-core), gibberellin (GARE-motif, P-box, TATC-box), salicylic acid (TCA-element), and jasmonate (CGTCA/TGACG motifs) were also identified, suggesting that *SbEXP* genes may be regulated by multiple hormonal pathways. Several stress-responsive elements were also detected, including MBS, DRE-core, LTR, STRE, TC-rich repeats, W box, WRE3, and WUN-motif. The enrichment of drought- and heat-responsive elements in several promoters suggests their potential involvement in stress responses. For example, the co-occurrence of MBS and ABRE elements in multiple *SbEXP* promoters indicates possible responsiveness to drought stress. Light-responsive elements such as G-box, Box 4, GT1-motif, and TCT-motif were also frequently observed, suggesting that *SbEXP* gene expression may be influenced by light-related signalling pathways. In addition, several cis-elements associated with plant growth and developmental processes were identified across *SbEXP* promoters. These elements, often associated with tissue-specific expression and developmental regulation, suggest that *SbEXP* genes may also participate in growth-related processes, including cell expansion, organ development, and differentiation. Overall, the diverse composition of cis-regulatory elements indicates that *SbEXP* genes are subject to complex regulatory control involving hormonal signalling, environmental responses, and developmental processes.

### miRNA-target interaction analysis of *SbEXP* genes

**Fig. 6:**
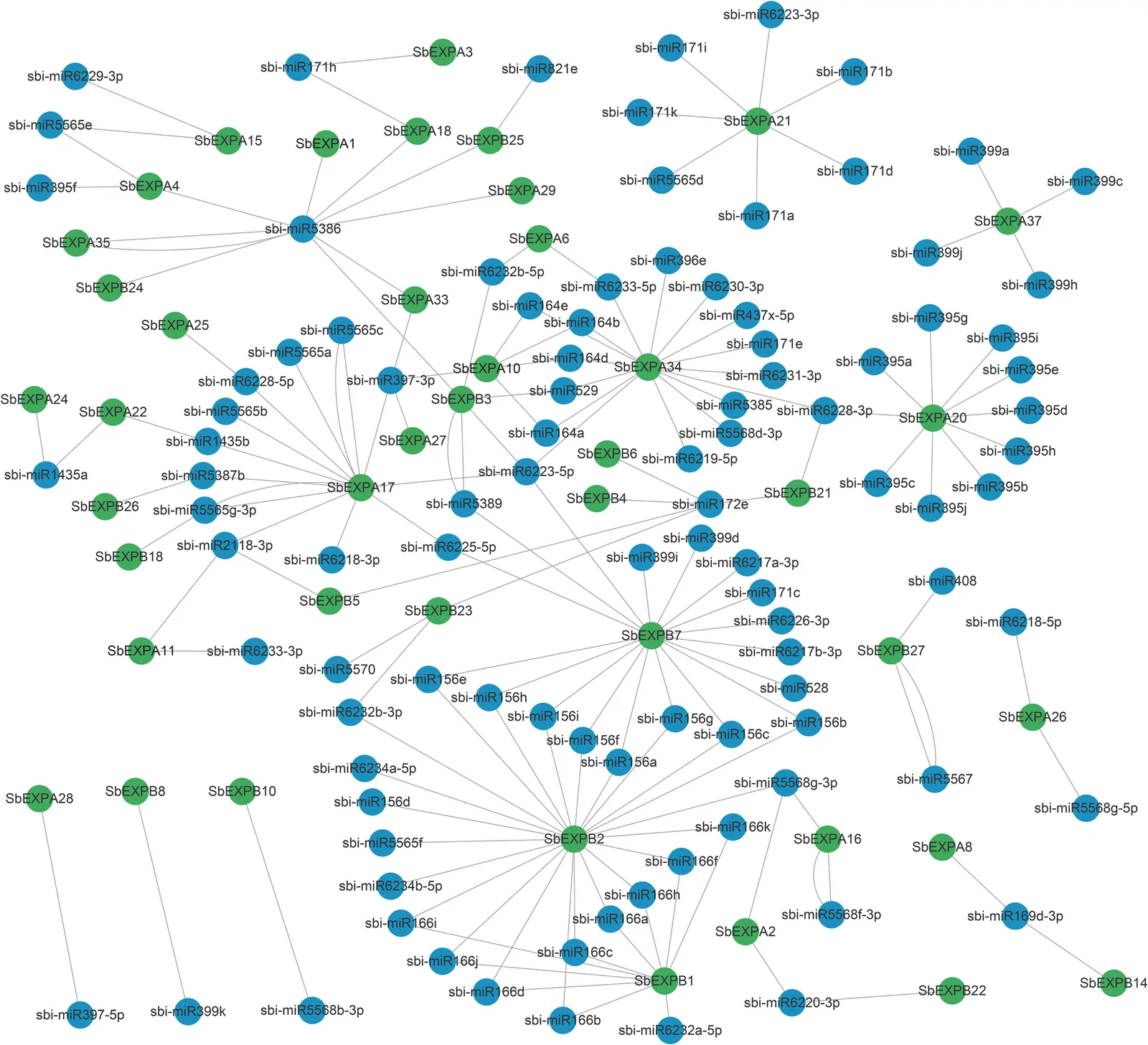
*SbEXP* genes miRNA-target interaction network in *Sorghum bicolor*. Links represent potential interactions between miRNAs and their targets

An in-silico analysis revealed multiple microRNAs (miRNAs) predicted to target *SbEXP* genes in *S. bicolor*. We identified 51 distinct miRNAs predicted to target *EXP* genes, yielding over 100 miRNA-mRNA pairs (Fig. 6; Supplementary Table S4). A majority of these interactions were predicted to result in mRNA cleavage, which is consistent with the high sequence complementarity typically observed in plant miRNA-mediated regulation. The miRNA-target interaction network revealed that several *SbEXP* genes are regulated by multiple miRNAs, indicating potential regulatory complexity. In particular, genes such as *SbEXPA17*,* SbEXPA34*,* SbEXPB2*, and *SbEXPB7* exhibited a high number of interactions, suggesting that they may function as central nodes in the regulatory network. A number of these miRNAs were also predicted to be multi-targeted, indicating simultaneous regulation of multiple *SbEXP* genes. For example, sbi-miR5386 was predicted to interact with several *EXP* genes from different subfamilies, whereas sbi-miR172 family members were predicted to interact with several *EXPB* genes. These observations suggest coordinated regulation of multiple *SbEXP* genes by specific miRNAs. The predicted miRNA targets were associated with relatively low expectation values, suggesting high-confidence targeting. In general, miRNA targets were found to be dispersed across the EXPA and EXPB subfamilies, suggesting that post-transcriptional regulation may be common for *SbEXP* genes. Overall, these findings suggest that *SbEXP* genes are subject to complex post-transcriptional regulation mediated by miRNAs, although experimental validation is required to confirm these predicted interactions.

### Protein-protein interaction (PPI) network analysis

**Fig. 7:**
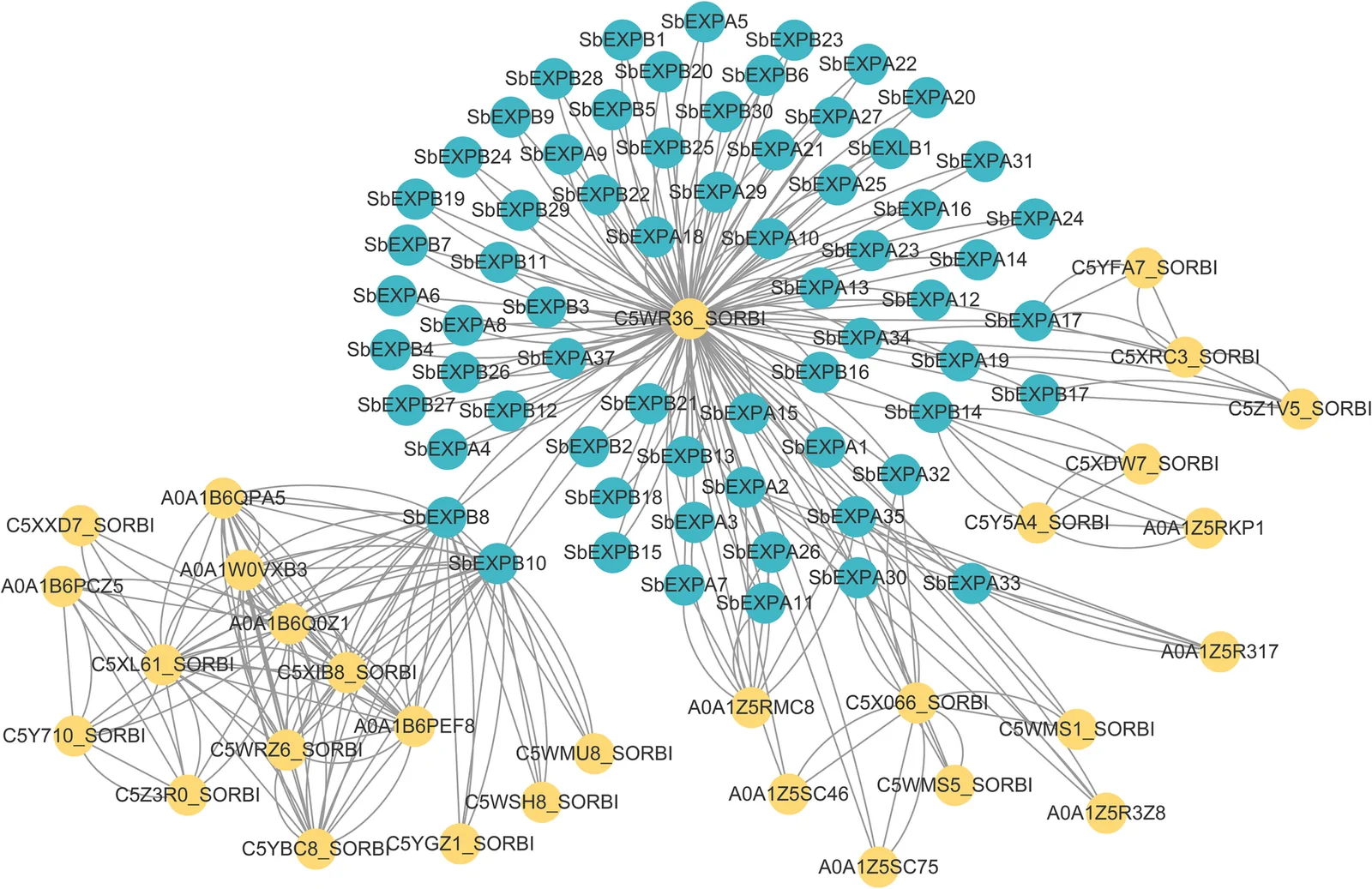
Network of protein-protein interactions (PPI) of SbEXP proteins in *Sorghum bicolor.* The network was built using the STRING database

To further explore the functional relationships among SbEXP proteins, a PPI network was predicted using the STRING database based on interaction data available for *S. bicolor*. The resulting network comprised 68 nodes and 232 edges, indicating relatively high connectivity among expansin proteins and their associated interactors (Fig. 7; Supplementary Table S5). Several SbEXP proteins, including SbEXPA17, SbEXPA34, SbEXPB2, and SbEXPB7, exhibited high connectivity, suggesting that they may function as central nodes within the predicted network. These hub genes may play important roles in coordinating expansin-related processes. Notably, a non-expansin protein, C5WR36_SORBI, exhibited numerous interactions with multiple SbEXP proteins, suggesting it may serve as a central interacting partner within the network. This observation suggests that EXPs may be integrated into broader protein interaction networks and may be involved in cell wall organization and remodeling. In addition, several SbEXP proteins were predicted to interact with proteins involved in cell wall modification, including enzymes involved in assembly and remodelling, further supporting their potential role in cell wall dynamics. Integration of the PPI network with miRNA interaction data revealed that several highly connected *SbEXP* genes, particularly *SbEXPA17*, *SbEXPB1*, and *SbEXPB2*, are also predicted targets of multiple miRNAs. This suggests a potential overlap between protein-level interactions and post-transcriptional regulation. Overall, the PPI network analysis suggests that SbEXP proteins may be part of complex interaction networks and may function cooperatively with other proteins in cell wall-related processes and potentially in stress responses, although experimental validation is required to confirm these interactions.

### Transcription factor (TF) regulatory network controlling *SbEXPs*

**Fig. 8:**
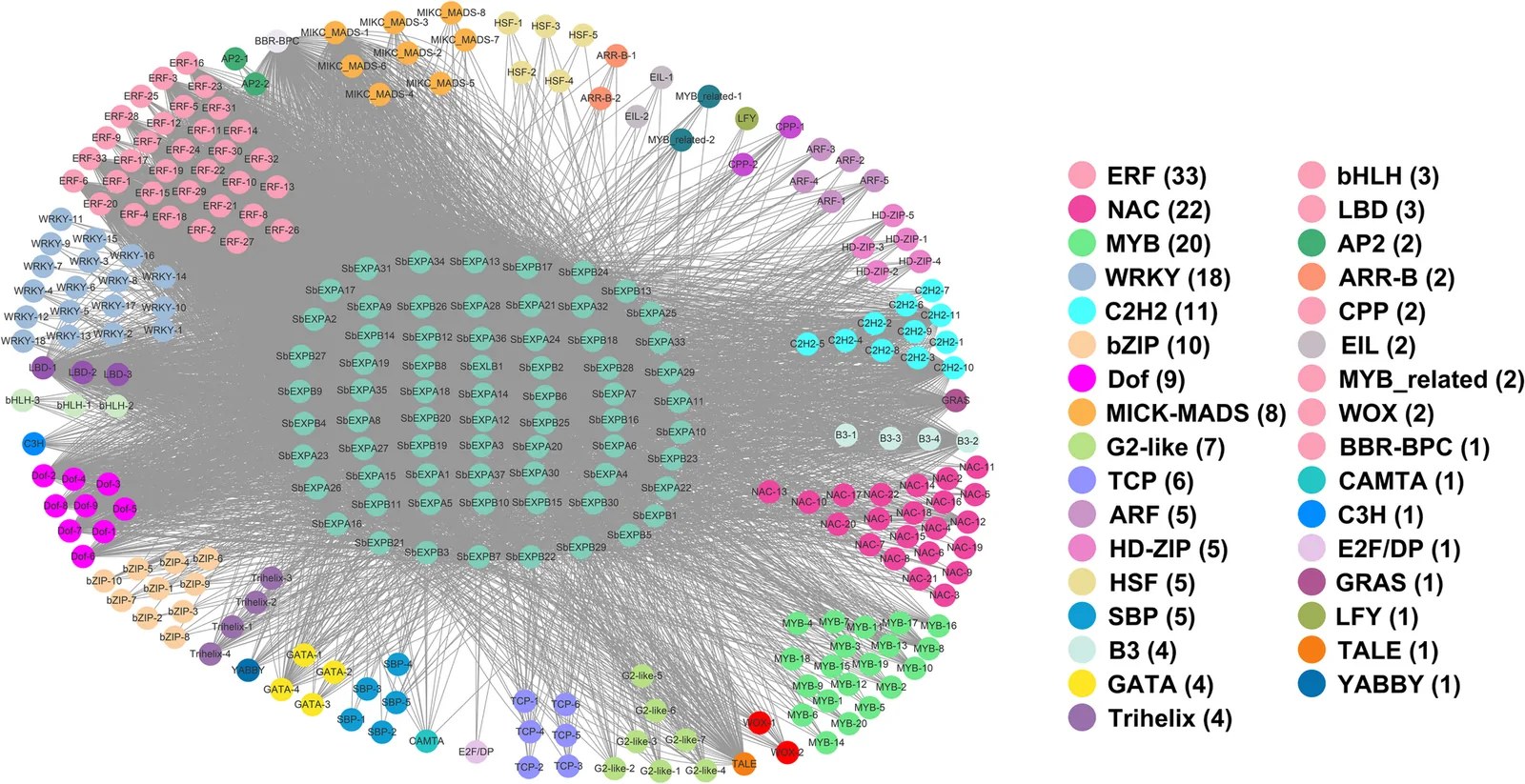
*SbEXP* genes transcription factor regulatory network in *Sorghum bicolor*. Network diagram depicting the predicted interactions between transcription factors and *SbEXP* target genes. TF families are represented by the color of the nodes

We investigated the regulatory network of *SbEXP* genes using a TF-target interaction network predicted from promoter analysis, as described in the Methods section. A total of 7,184 TF binding events were predicted, involving 202 TFs and 68 *SbEXP* genes (Fig. 8; Supplementary Tables S6 and S7). TF binding sites were identified on both DNA strands, ranging from 7 to 28 bp, with high matching scores, indicating reliable prediction of TF-target interactions. The predicted TFs belonged to diverse families, many of which are known to be associated with stress responses, hormonal signaling, and developmental processes. Major TF families included ERF (33 TFs), NAC (22), MYB (20), WRKY (18), C2H2 (11), bZIP (10), and Dof (9), along with additional families such as MIKC-MADS, G2-like, TCP, ARF, HD-ZIP, HSF, SBP, B3, GATA, bHLH, LBD, WOX, and YABBY. The predominance of these TF families suggests that *SbEXP* genes may be regulated by multiple signalling pathways related to environmental responses and development. The TF regulatory network showed considerable variation in the number of TF interactions among *SbEXP* genes. Several genes, including *SbEXPA1*, *SbEXPA12*, *SbEXPA17*, *SbEXPA34*, and *SbEXPB7* exhibited a high number of TF interactions, suggesting that they may act as central nodes within the regulatory network. These genes may play important roles in integrating signals from different regulatory pathways. Overall, the TF network analysis indicates that *SbEXP* genes are likely subject to complex transcriptional regulation involving multiple TF families, although these interactions are based on computational predictions and require experimental validation. Notably, the extensive interactions of *SbEXPA17* with ERF, NAC, MYB, and Dof TFs suggest its potential role in integrating multiple signaling pathways associated with stress and development. Taken together, these results suggest that *SbEXP* genes are part of a complex TF-mediated regulatory network. Overall, these interactions provide insight into the potential regulatory mechanisms controlling *SbEXP* gene expression, particularly in relation to stress-responsive and developmental processes.

### Expression profiling of *SbEXP* genes across tissues and stress conditions

**Fig. 9:**
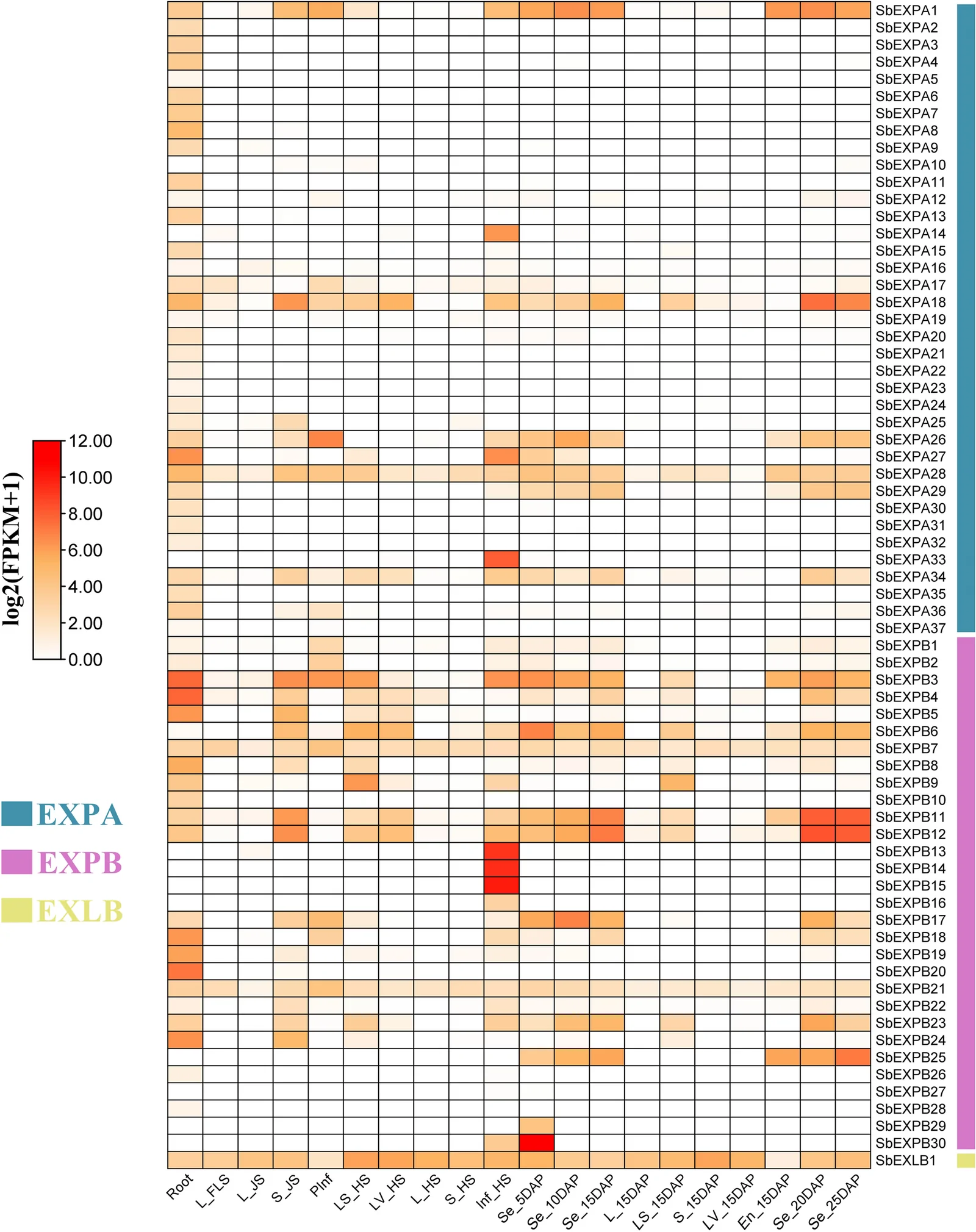
Expression profiles of *SbEXP* genes in various tissues at various developmental stages in *Sorghum bicolor*. The expression profiles of each *SbEXP* were presented as log2(FPKM + 1) values. The color bar represents expression value intensity

To gain insight into the potential roles of *SbEXP* genes, we analyzed their expression patterns across tissues and during abiotic stress conditions using publicly available RNA-seq data. The expression levels were transformed to log₂(FPKM + 1) and represented as heatmaps (Fig. 9; Supplementary Table S8). The *SbEXP* genes exhibited diverse expression patterns across tissues, indicating functional differentiation within the gene family. Several EXPB genes showed relatively higher expression in reproductive tissues, such as seeds and panicles, whereas many EXPA genes were more highly expressed in vegetative tissues, including roots, stems, and leaves. This distribution is consistent with the known roles of EXPs in cell expansion and organ development. In addition, some *SbEXP* genes displayed constitutive expression across multiple tissues, while others showed strong tissue specificity, highlighting potential specialization of individual gene members. Under abiotic stress conditions, several *SbEXP* genes exhibited differential expression patterns. Some genes were upregulated under salt and heat stress, while others showed moderate or low changes in expression, indicating variable responsiveness among family members. Overall, the combined tissue-specific and stress-responsive expression patterns suggest that *SbEXP* genes may contribute to both developmental processes and adaptive responses to environmental stress, potentially through modulation of cell wall dynamics.

### Experimental validation of the *SbEXP* gene expression under drought stress

**Fig. 10:**
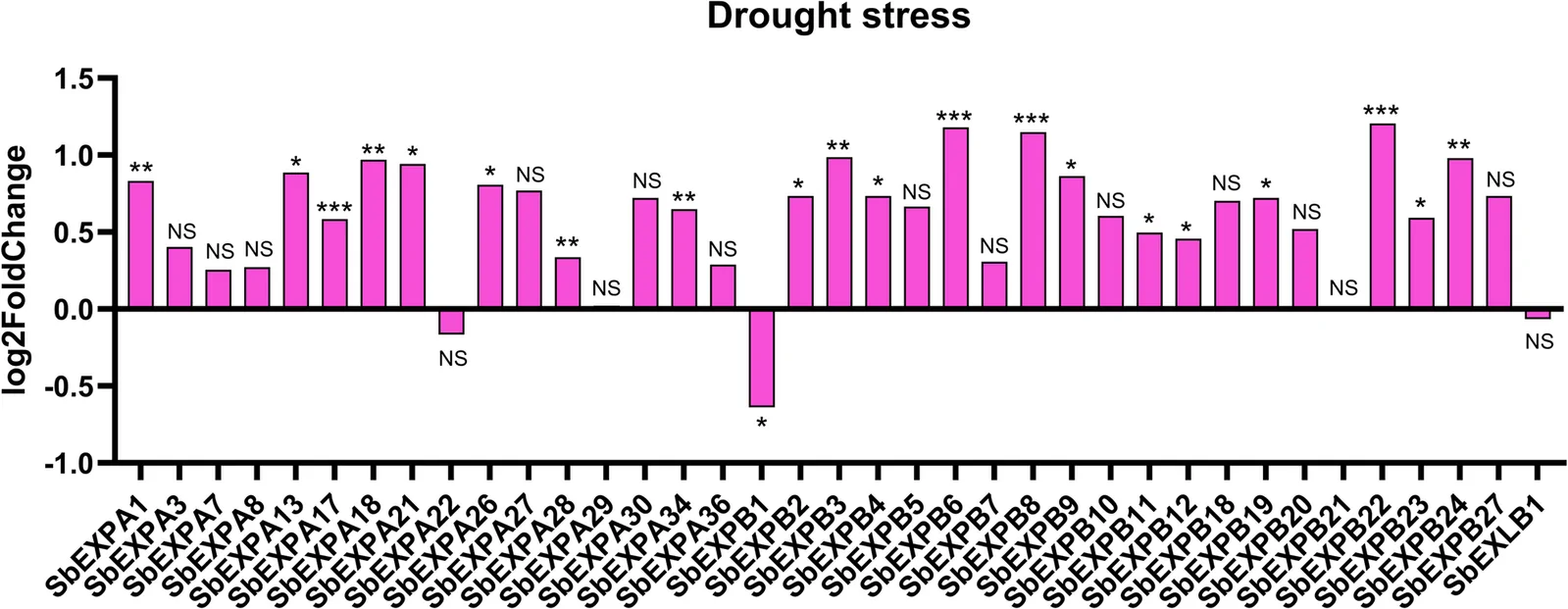
*SbEXPs* expression profile under drought stress. Bar plot illustrating log2 fold-change in the expression of *SbEXP* genes during drought. Positive values indicate upregulation, whereas negative values indicate downregulation relative to the control (CK) values. Significance levels of 0.05 (*), 0.01 (**), 0.001 (***), and non-significant differences (NS) are used to indicate statistical significance

**Fig. 11:**
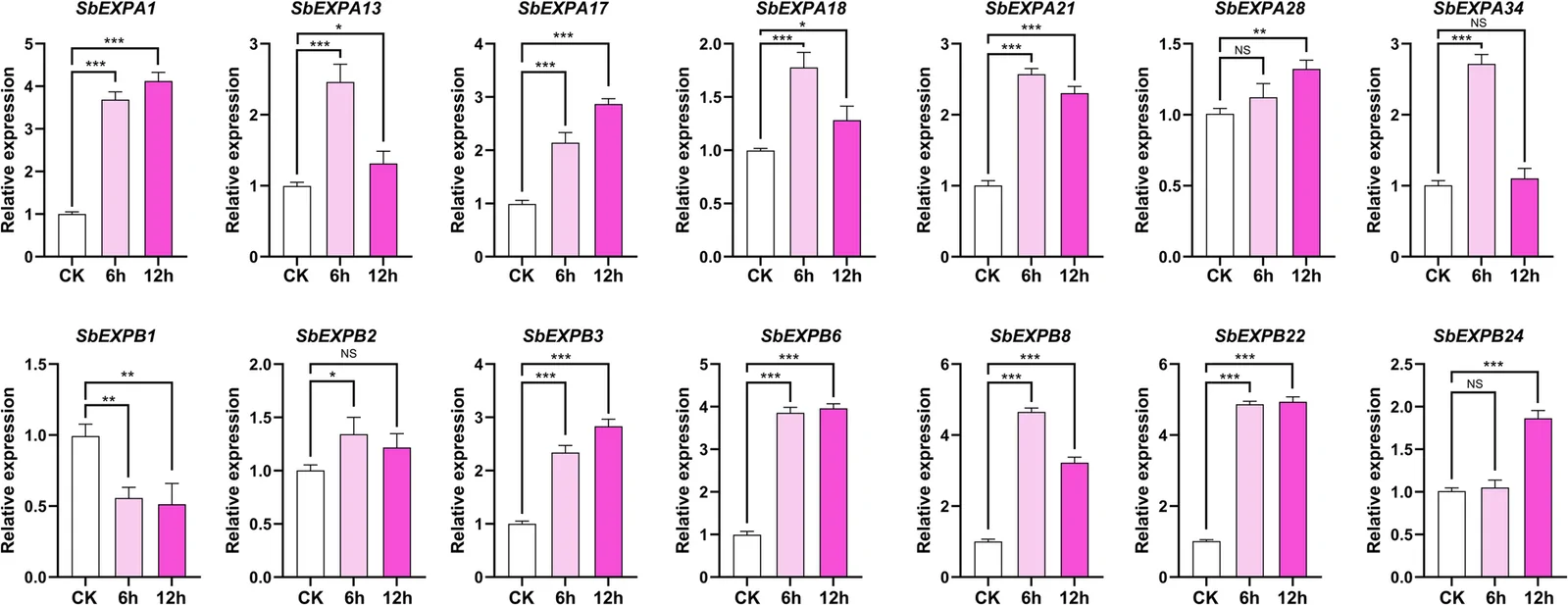
qRT-PCR confirmation of the chosen genes of *SbEXP* in drought in *Sorghum bicolor*. Relative expression level of *SbEXP* genes under drought conditions of 0 h (CK), 6 h, and 12 h is presented, with statistical significance marked (*p < 0.05, **p < 0.01, ***p < 0.001)

To determine the responsiveness of *SbEXP* genes to drought stress, we examined their expression profiles via RNA-seq and validated them by qRT-PCR. To assess the expression profiles under drought stress, public RNA-seq data from GSE128441 were used, and fold changes were calculated as log₂ values (Fig. 10; Supplementary Table S9). The RNA-seq analysis revealed that many *SbEXP* genes exhibited differential expression under drought stress. Several members of both EXPA and EXPB subfamilies were markedly upregulated, whereas others showed moderate induction or downregulation, indicating diverse transcriptional responses within the gene family. Notably, a subset of genes displayed consistent upregulation across conditions, suggesting potential involvement in drought response. To validate these findings, selected *SbEXP* genes were analyzed by qRT-PCR under polyethylene glycol (PEG)-simulated drought conditions (Fig. 11). The qRT-PCR results were largely consistent with the RNA-seq data, supporting the reliability of the transcriptomic analysis. For example, *SbEXPA17*, *SbEXPB6*, and *SbEXPB8* showed significant induction at both 6 h and 12 h compared to the control (0 h), indicating strong responsiveness to drought stress. Temporal expression patterns further revealed that some genes maintained or increased their expression over time, suggesting sustained transcriptional activation under stress conditions. However, not all *SbEXP* genes responded similarly, highlighting functional diversity within the gene family. Overall, the combined RNA-seq and qRT-PCR analyses identify a subset of *SbEXP* genes that are strongly responsive to drought stress, suggesting their potential involvement in stress adaptation, possibly through modulation of cell wall properties.

## Discussion

EXPs are also well known for their role in regulating cell wall stretchability and the associated processes in plant development, growth, and stress adaptation [42]. EXPs enable cell expansion by relaxing cellulose-hemicellulose networks and play roles in a range of physiological activities, including root and organ development and stress responses [15, 16]. In this study, we conducted a comprehensive genome-wide analysis of the *SbEXP* gene family in *S. bicolor*, including evolutionary, regulatory, and expression profiling, as well as experimental verification. Such integrative genome-wide analyses have been widely applied to characterize diverse plant gene families, providing valuable insights into their evolutionary relationships, regulatory mechanisms, and potential biological functions [43–45]. Our findings will provide improved insight into the structural variability and significance of EXPs in sorghum, particularly under drought stress.

*EXP* genes are classified into four subfamilies: EXPA, EXPB, EXLA, and EXLB [21]. Consistent with previous studies, the *SbEXP* gene family was predominantly composed of EXPA and EXPB members, while the absence of EXLA members and the presence of only one EXLB gene suggest lineage-specific diversification in monocots [8, 46]. Gene duplication analysis indicated that both tandem and segmental duplications contributed to the expansion of the *SbEXP* gene family, with segmental duplications playing a more prominent role. Similar evolutionary patterns have been reported in other plant species, where duplication events contribute to gene family expansion and functional diversification [12, 47, 48]. Additionally, SbEXP proteins retained conserved DPBB_1 and expansin_−_C domains, which are essential for cell wall binding and loosening functions [49, 50], whereas variations in gene structure and motif composition may contribute to functional diversification.

Regulatory analyses revealed that *SbEXP* genes are subject to multiple layers of control, including cis-regulatory elements, transcription factors, and miRNAs. Promoter analysis showed enrichment of stress- and hormone-responsive elements, such as ABRE and MBS, which are associated with ABA signaling and drought responses [51–53]. MBS, which are associated with ABA signaling and drought responses. Furthermore, transcription factor analysis predicted interactions with TF families including ERF, NAC, MYB, WRKY, and bZIP, which are widely implicated in abiotic stress responses and hormone-mediated regulation [54, 55]. In addition, miRNA target prediction suggested potential post-transcriptional regulation of *SbEXP* genes, consistent with findings in other plant species such as rice and sugarcane [56–58]. It should be noted that these TF and miRNA interactions are based on computational predictions and require experimental validation. Collectively, these results suggest that *SbEXP* genes may be integrated into complex regulatory networks involved in plant development and stress responses.

Expression profiling of *SbEXP* genes across tissues revealed substantial variation, indicating functional diversification within the gene family. Tissue-specific expression patterns suggest roles in developmental processes, consistent with observations in sweet potato [21], potato [51], wheat [59], sugarcane [58], and tea [60]. The increased expression of some genes in highly active tissues is consistent with their roles in cell growth and development [16].

Abiotic stress treatments altered the expression patterns of several *SbEXP* genes, suggesting their potential involvement in stress responses. EXPs have been reported to contribute to stress tolerance by regulating cell wall properties and maintaining cellular integrity under stress conditions [14]. In this study, RNA-seq analysis of the GSE128441 dataset [38] and qRT-PCR validation showed that several *SbEXP* genes were differentially expressed under drought stress. For example, *SbEXPA1*7, *SbEXPB6*, and *SbEXPB8* exhibited dynamic expression patterns under PEG-induced drought conditions, indicating their potential responsiveness to water-deficit stress. Similar expression patterns have been reported in other plant species, such as *Moso bamboo* [55] and *Wild Arachis* [56], where EXPs are associated with stress adaptation through cell wall modification [61]. However, not all *SbEXP* genes were induced under drought conditions, as some exhibited reduced or unchanged expression levels, suggesting functional diversity within the gene family and context-dependent regulatory roles.

Taken together, these findings suggest that *SbEXP* genes may contribute to drought adaptation by modulating cell wall plasticity. Under drought stress, maintaining cell wall flexibility is essential for sustaining cell growth and preventing structural damage [59, 62]. EXPs are likely associated with this process by facilitating cell wall loosening [14, 63]. This function may be coordinated with hormone signaling pathways, particularly abscisic acid (ABA), which plays a central role in drought responses [64].

Overall, this study provides a comprehensive characterization of the *SbEXP* gene family in *S. bicolor*, offering insights into its evolutionary features, regulatory networks, and potential involvement in drought response. Candidate genes such as *SbEXPA17*, *SbEXPB6*, and *SbEXPB8* represent promising targets for further functional investigation. Given the growing importance of improving drought resilience in crops under changing climatic conditions, these findings may inform future efforts to enhance stress tolerance in sorghum. Future studies should focus on functional validation of key genes using transgenic or gene-editing approaches, as well as elucidating their interactions with other stress-related pathways. In addition, examining EXP function across diverse developmental stages and environmental conditions will help to further clarify their biological roles.

## Conclusion

This study provides a comprehensive characterization of the EXP gene family in *S. bicolor*, identifying 68 members predominantly belonging to the EXPA, EXPB, EXLA, and EXLB subfamilies. Structural and evolutionary assessments indicate conserved domain organization, with gene family expansion likely driven by duplication events. Regulatory analyses suggest that *SbEXP* genes are subject to complex, multi-layered control involving diverse cis-regulatory elements, transcription factors, miRNAs, and protein-protein interactions, linking their regulation to both developmental and environmental processes. Network analyses highlight key genes, including *SbEXPA17*,* SbEXPB1*, and *SbEXPB2*, as potential central nodes within these regulatory frameworks. Expression profiling, supported by experimental validation, demonstrates that several *SbEXP* genes are responsive to drought stress, particularly *SbEXPA17*,* SbEXPB6*, and *SbEXPB8*, indicating their potential roles in stress adaptation. Collectively, these findings provide a systematic understanding of the structural features, regulatory mechanisms, and expression dynamics of *EXP* genes in sorghum and identify promising candidates for future functional studies and crop improvement.

## Supplementary Information

Supplementary Material 1: Table S1. Pfamscan results. Table S2. The detailed information on cis-acting analysis among *SbEXPs* in Sorghum. Table S3. qPCR primers. Table S4. miRNA targets. Table S5. Protein-protein interaction. Table S6. TFs regulation prediction. Table S7. TFs enrichment results. Table S8. Expression pattern of *SbEXPs* in various tissues. Expression presented as log2(FPKM+1) values. Table S9. Expression pattern of *SbEXPs* in drought. Expression presented as log2foldchange values.

## Data Availability

All data generated or analyzed during this study are included in this published article.
